# Quantitative Study of Predicting the Effect of the Initial Gap on Mechanical Behavior in Resistance Spot Welding Based on L-BFGS-B

**DOI:** 10.3390/ma17194746

**Published:** 2024-09-27

**Authors:** Yulong Su, Kai Song, Zhanpeng Du, Kangchen Yu, Zhaohui Hu, Hao Jin

**Affiliations:** 1 State Key Laboratory of Advanced Design and Manufacturing Technology for Vehicle, Hunan University, Changsha 410082, China; syl_0109@163.com (Y.S.); 18757837488@163.com (K.Y.); hzh811202@163.com (Z.H.); 2 School of Automotive and Traffic Engineering, Jiangsu University, Zhenjiang 212013, China; dzp1014@163.com; 3 Guangxi Liuzhou LEGN Technology Co., Ltd., Liuzhou 545000, China; j1mp_1996@163.com

**Keywords:** resistance spot welding, initial gap, prediction model, mechanical behaviors, L-BFGS-B, finite element model

## Abstract

The initial gap (IG) is frequently occurring in the process of resistance spot welding (RSW) for automotive body-in-white structures. It is an inevitable challenge that the RSW with IG can negatively impact the welding quality, subsequently reducing the structural integrity and safety of the vehicle. This research aims to study the influence of the IG on RSW mechanical behaviors based on the refined finite element model (FEM) of RSW with different IGs under tensile shear load. The influence of six types of IGs on the peak load and fracture modes of RSW of plates with similar thicknesses of 1.0 mm and 1.5 mm is investigated through FEM and experiments. To quantify the influence of the IG on the RSW’s deformational behavior, a prediction model is introduced to predict the peak load of RSW with different IGs. The prediction model is locally optimized through the Limited memory Broyden Fletcher Goldfarb Shanno (L-BFGS-B) optimization algorithm. Based on the prediction model, the relationship among the peak load values of the tensile shear specimens, the IG, and the mechanical behavior of the RSW is revealed. The results show that the IG has an obvious influence on the peak load values of RSW under tensile shear load, and the fracture modes are both the pull-out fracture (PF) mode. The peak load values of the RSW’s tensile shear specimens are decreased with the increment in the IG. Finally, the prediction model can accurately predict the peak load for various IGs, with errors of no more than 3%.

## 1. Introduction

RSW is the dominant sheet joining process in automotive body-in-white structures [1,2,3] due to its significant cost advantage and high-efficiency characteristics [4,5]. Generally, a modern steel vehicle body structure contains 3000~6000 RSWs, which account for approximately 90% of the total welding workload [6,7]. The rapid heating of RSW can lead to the forming rebound of parts and poor assembly, resulting in frequent changes in the welding state, especially in the large-scale automobile body production lines [8]. Some researchers have tried to reduce such fluctuations by changing the parameters of RSW—for example, Kas, Z. et al. [9] designed an AOSA controller for RSW to compensate for some process parameter changes. In the process of high-speed mass production, the wear and assembly changes of mechanical parts can inevitably lead to abnormal welding, in which the initial plate gap is one of the problems [10,11]. Once these interference factors appear, the contact area and resistance characteristics of the welding process are changed, resulting in significant fluctuations in the weld quality and even welding defects [12]. Common welding defects resulting from these disturbances including inadequate venting and diminished weld size, both of which can substantially compromise the overall quality and strength of the weld [13].

Throughout the normal driving process of the vehicle and the event of a crash, RSWs consistently endure diverse and varying degrees of external forces [14]. Therefore, in the manufacturing of automobile bodies, the issue of gaps between welded sheets is a prevalent defect that can not only lead to misalignment during assembly but also negatively impact the overall quality of the welding process [10,15]. In addition, the stamping deviation of thin plate structural parts, process clamping errors, and other errors are accumulated in the body assembly process, forming a comprehensive deviation of body manufacturing. IG also occurs between the thin plates. Although the manufactured components exhibit high precision, geometry alterations related to the process may still result in gaps between the welded sheets [16]. In RSW’s process, the IG between workpieces is regarded as a critical factor contributing to substantial deformation during the assembly process [17]. Yurci, C. et al. [18] also highlight that the deformation occurring post-welding can substantially influence the tolerance chain and precision of the component. It indicates that when the plate is engaged in the subsequent welding position, an IG between the plates has already occurred due to the preceding welding. The IG between the plates is an inevitable problem before the welding process in the RSW of thin plates. Thus, research on the influence of the IG on the mechanical characteristics and failure behaviors of RSW is necessary. When the IG increases, the surrounding area of RSW experiences more significant plastic deformation. In order to further investigate this phenomenon, Murakawa et al. [19] comprehensively investigated a variety of deformation factors including geometric and material nonlinear deformation. Some researchers have proven that the IG between mild steel has a great effect on nugget formation and expulsion by changing the contact between sheets and electrodes. The expulsion possibility increases with the increase in the IG [20]. Min [21] states that the presence of IGs reduces the area of contact between the sheets, leading to a reduction in the size of the fusion zone (FZ) and inhibiting the thermal expansion effect of the workpiece during welding. Podrzaj et al. [10] also observed the shrinking of the nugget diameter and demonstrated that the presence of IG could reduce the electrode force between the two sheets. Due to the long time that is set for the cooling process in the simulation, Shen et al. [22] obtained a relatively stable size of the FZ. They [23] also showed that with the increase in the plate thickness and IG, the expulsion can be effectively suppressed by increasing the electrode force during the process of RSW for DP steel. Xia et al. [24] discussed the effect of IG on the formation and expansion of FZ and the internal mechanism of the welding process signal.

At present, the mechanical properties of RSW in the presence of IG are not widely and deeply studied. Yang et al. [25] pointed out that the presence of IG has no significant impact on the strength of RSW. Zhou et al. [3] obtained different results. They found that the IG and edge proximity conditions reduce the diameter of the FZ and the strength of the RSW. During the tensile shear experiments, the specific combination of the IG between the plates and the plate angle leads to the formation of small and asymmetrical FZ, which in turn adversely affects the peak load [26]. Ashadudzzaman et al. [27] discussed improving resistance spot welding quality by incorporating adaptive control systems to adjust process disturbances such as gaps. However, there are few quantitative studies on the effect of the IG on the deformation failure behaviors of RSW. To better understand this problem and give a comprehensive explanation, more detailed research is needed. As an efficient and widely used method, FEM has shown remarkable advantages in the modeling process of RSW and the analysis of mechanical deformation characteristics [28]. Zhang et al. [29] used the finite element analysis technology to deeply explore the effective control of expulsion and the influence on the formation of FZ size during the RSW of DP steel plates with IG. Mikno et al. [30] studied the shear force characteristics and their related mechanism during static tensile experiments of the RSW by applying three-dimensional FEM. The 3D-refined FEM of RSW with the presence of the IG built by Su et al. [31] based on an equivalent displacement method can predict the mechanical properties well. So, this paper is based on the refined 3D FEM for RSW with the IG to study the influence of IG on the mechanical performance of RSW.

Above all, the failure behaviors of RSW with the IG should be investigated. The quantified influence of the IG on the failure behaviors of RSW should also be a research focus. Low-carbon steel materials are still widely used in automotive body-in-white (BIW) structures due to their good weldability and low cost [32]. Therefore, low-carbon steel is selected as the material for RSW specimens. The effect of the IG on the RSW mechanical behaviors based on the refined finite element model (FEM) of RSW with an IG under tensile shear load is investigated. In this paper, six types of IGs are chosen for RSW, ranging from 0 mm to 5 mm, with a gap increment of 1 mm. The IG exhibits a significant effect on the maximum bearing capacity of RSW under tensile shear load. With the increment in IGs, the dominant RSW fracture modes are both PF modes. Moreover, a prediction model is introduced for the plate thicknesses of 1.0 mm and 1.5 mm, respectively. The L-BFGS-B is a robust algorithm that can find a locally optimal solution in a relatively small number of iterations. It seeks the local optimal solution through the stepwise approximation method, which is especially suitable for the case with complex objective functions. Therefore, the prediction model is locally optimized through the L-BFGS-B optimization algorithm. Based on the established prediction model, the internal relationship among the peak load of the tensile shear specimens, the IGs, and the mechanical behavior of the RSW is quantified. The peak load values of RSW with different IG under tensile shear loads can be accurately predicted, and it can offer theoretical guidance for the prediction of the mechanical properties of RSW with such an IG.

## 2. Material and Methods

### 2.1. Experimental Details

**Figure 1:**
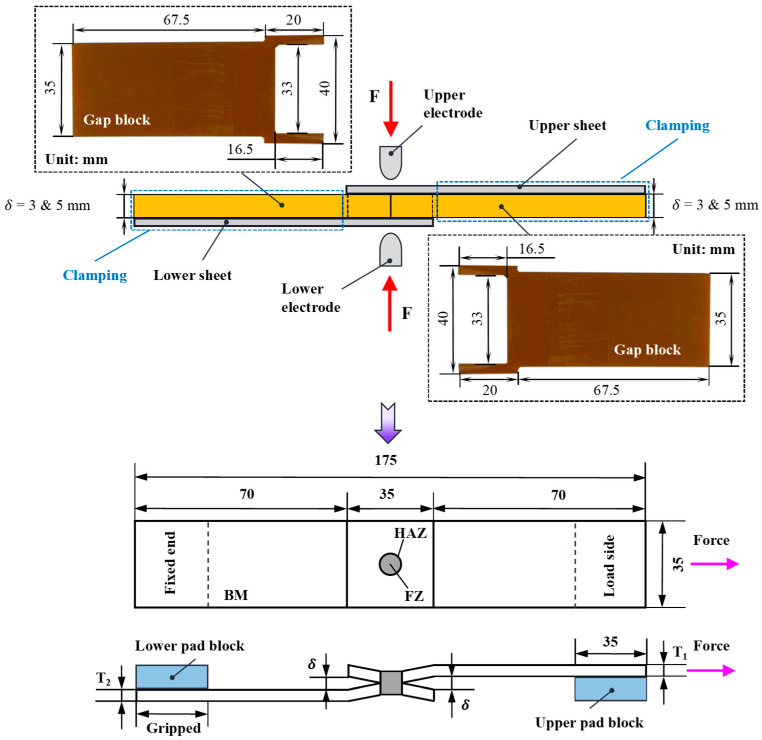
The assembly diagram and dimensional diagram of RSW’s tensile shear specimen with IG.

In the study, low-carbon steel is still widely used for the automobile BIW [32]. Therefore, it is selected as the material for RSW specimens. The low-carbon steel sheets are welded as tensile shear specimens to research the mechanical properties and failure model. Load displacement curves and the peak load can be obtained by quasi-static tensile experiments. The mature welding process parameters (weld time: eight cycles, electrode force: 1.6 kN, weld current: 6.56 kA, weld voltage: 0.8 V~1.2 V) are selected to make RSW’s specimens accomplished by a pneumatic spot-welding machine (6.4 mm electrode tip diameter). The sheet thickness and IG of the resistance spot welding experimental samples in this paper are shown in Table 1. The desired specimens of RSW with IGs are made by the weld gap blocks. The thickness of a gap block is equal to the IG. The weld gap blocks are placed in the middle of the upper and lower plates, as shown in Figure 1. Once the welding is complete, the gap blocks can be pulled out from both sides. In the welding process, the current passes through the plate and the electrode contact area, and the resistance heating melts the contact surface to achieve the connection. The welding gap blocks are made of non-conductive and high-hardness bakelite board to ensure no deformation under pressure and guarantee welding quality. Detailed geometric information of the tensile shear specimen is shown in Figure 1. In the tensile experiment, to ensure that the test specimens of RSW can withstand the resultant force acting along the centerline of the RSW, both ends of the pad block are incorporated into the specimens. The pad block’s thickness is the sum of the IG and the thickness of the other sheet.

**Figure 2:**
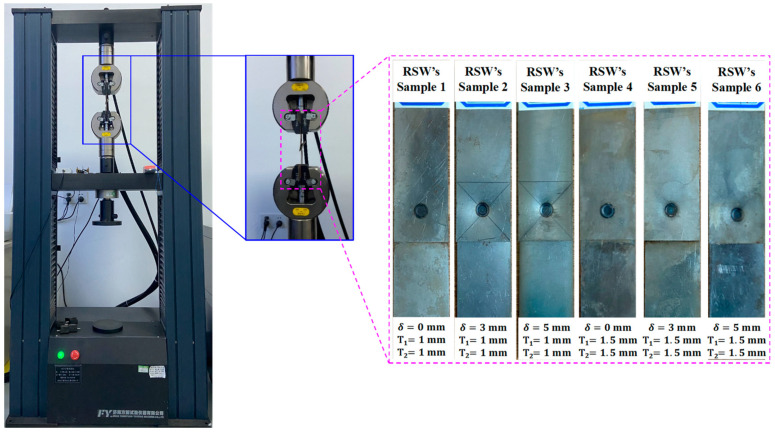
Tensile test of RSW’s tensile shear specimens with three IGs.

**Figure 3:**
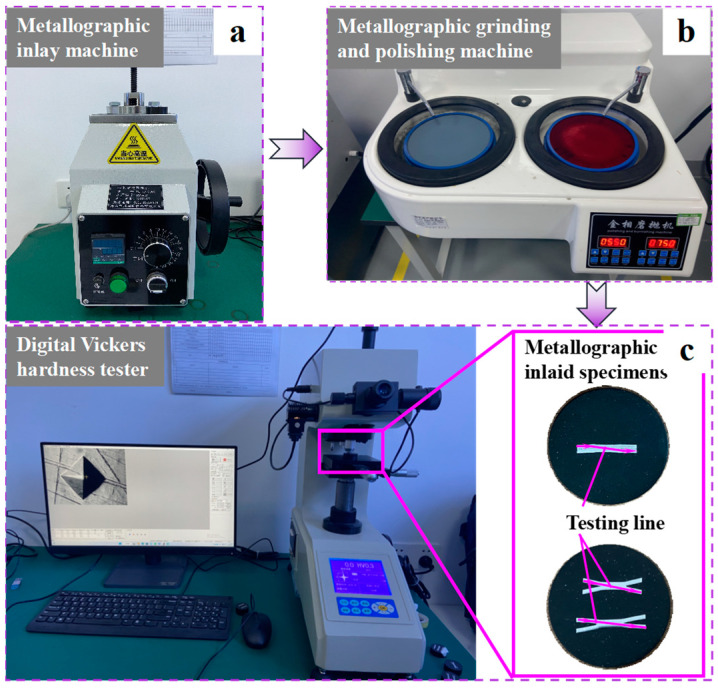
Hardness test of RSW’s specimens; (**a**) Metallographic inlay machine; (**b**) Metallographic grinding and polishing machine; (**c**) Metallographic inlaid specimens.

To evaluate and analyze the deformation behaviors of RSW with IGs, the tensile shear specimens are made by the national standard “GB/T228-2002: Metal materials room temperature tensile test method”, with a tensile speed of 2 mm/min. The quasi-static electronic universal testing machine and the specimens of RSW with three IGs are shown in Figure 2. The Vickers hardness of each zone of RSW is also obtained by an HV-1000 automatic turret digital Vickers hardness tester (Jinan FangYuanShiYanYiQi Co., Ltd., Jinan, China), as shown in Figure 3. The specific test parameters include an applied load of 300 g, hold time of 10 s, and measurement step set to 0.5 mm.

### 2.2. FEM for RSW with Different IGs

**Figure 4:**
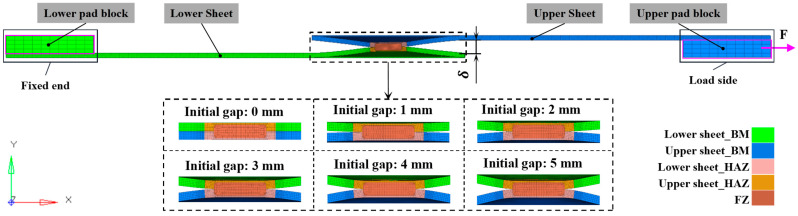
FEM of RSW with different IGs.

The FEM used in this study is designed to capture the mechanical performance and failure model. The refined FEM based on an equivalent displacement method can accurately simulate the complex mechanical behaviors for the RSW with an IG subjected to tensile shear loading [31]. This model can accurately reflect the failure deformation behavior of RSW with IGs, which provides strong support for further research. Therefore, this method is adopted to establish the refined FEM of RSW’s tensile shear specimens, as shown in Figure 4. The area of RSW is subdivided into the FZ, heat-affected zone (HAZ) and base metal (BM). In terms of boundary conditions, the fixed end is set to a fully constrained state. On the side where the load is applied, all the degrees of freedom except the translation degrees of freedom along the X axis are constrained, simulating the loading conditions in the real experiment. In terms of material properties, the MAT24 is adopted in LS-DYNA, and its elastic modulus *E* is set as 203 GPa, its Poisson’s ratio is set as 0.3, and its density is set as 7.85 × 10^−9^ kg/m^3^. In this study, six types of IGs are chosen, ranging from 0 mm to 5 mm, with a gap increment of 1 mm, as described in Figure 4.

## 3. Results

### 3.1. Hardness of RSW

**Figure 5:**
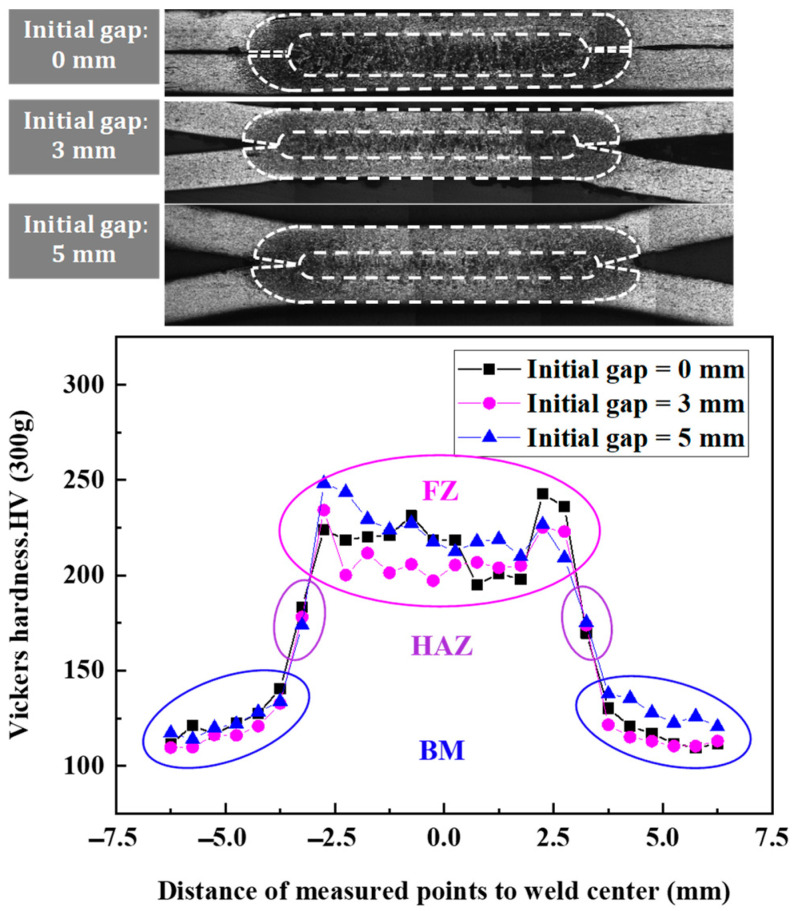
RSW’s hardness varies with three different IGs and the metallography of the RSW cross-section.

**Figure 6:**
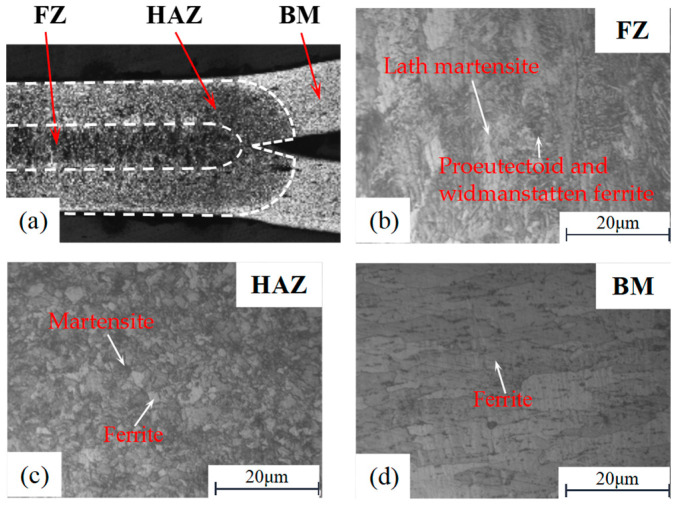
The scanning electron microscope image for three zones of RSW; (**a**) Half cross-section of RSW; (**b**) The microstructure of FZ; (**c**) The microstructure of HAZ; (**d**) The microstructure of BM.

The hardness distributions of the RSW with three different IGs of tensile shear specimens are shown in Figure 5. The hardness values fluctuate between 195 and 250 HV in the FZ of RSW with three IGs. It is the largest in the three zones of the RSW, whose microstructure is mainly composed of lath martensite (as shown in Figure 6b), which shows columnar characteristics, and on this basis, it also contains a certain proportion of proeutectoid and Widmanstätten ferrite (as shown in Figure 6b), which together constitute the complex microstructure of this region [33]. However, the hardness values of the HAZ decrease rapidly due to the combination of the high heating temperature and slow cooling conditions, whose microstructure is mainly composed of fine martensite and ferrite, as shown in Figure 6c. It occurs when the microstructure gradient and the original transformation are altered [33]. The hardness value of HAZ is around 175 HV. The BM microstructure consists of ferrite grains (as shown in Figure 6d) with carbides situated at grain boundaries, resulting in the hardness of BM being the lowest in the three zones of the RSW. The hardness values of the BM fluctuate between 100 HV and 140 HV.

**Figure 7:**
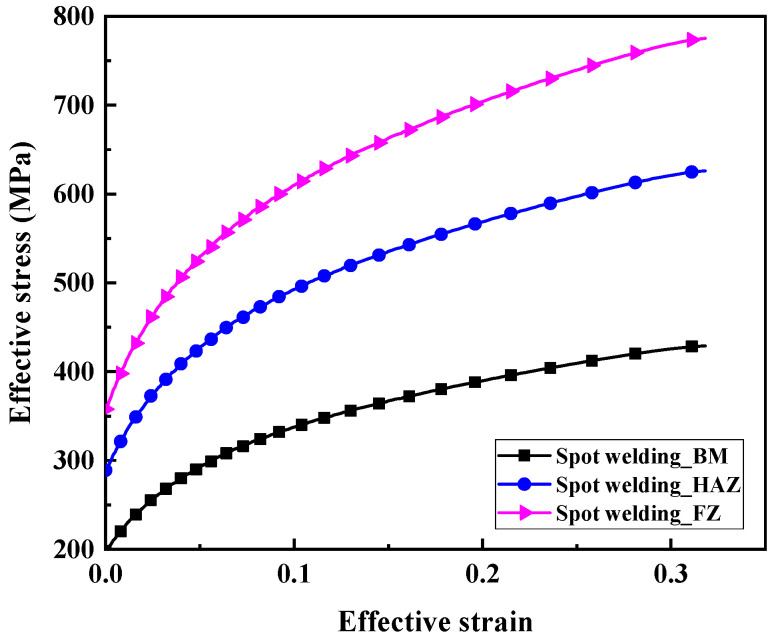
Effective stress–strain curves for three zones of RSW.

The average hardnesses in the three zones of the RSW with three different IGs are very similar, as shown in Figure 5. This indicated that the effect of the IG on the hardness in the three zones of the RSW is almost minor. Therefore, the average hardness values of the FZ, HAZ, and BM are adopted in this study, as shown in Table 2. Then, utilizing the real stress–strain curves of BM in RSW acquired through tensile testing, the real stress–strain curves for both the FZ and HAZ in RSW are derived and established by employing an approximate fitting technique, as described in Figure 7. The fitting formulas adopted in this paper have been published in the literature [31]. Finally, the real stress–strain mechanical parameters for the three zones are assigned to the corresponding zone in the FEM of RSW (discussed in Section 2.2).

### 3.2. Peak Loads of Tensile Shear Specimens

**Figure 8:**
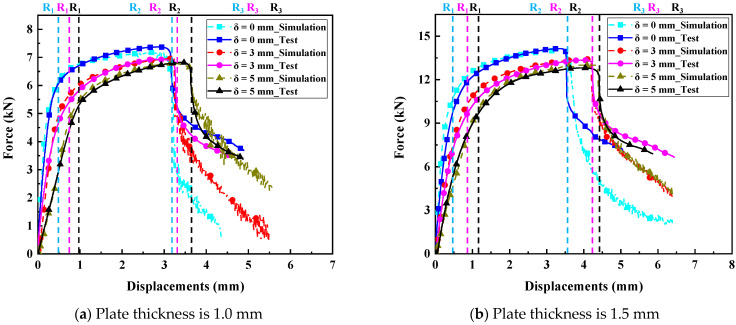
The comparison of the force–displacement curves of RSW with three different IGs acquired from the simulation and test.

As mentioned above, two types of plates with similar thicknesses are welded with three different IGs (0 mm, 3 mm, and 5 mm), as shown in Table 1. The force and displacement curves are acquired from the simulation and experiments of RSW with three different IGs, as shown in Figure 8. They show that the FEM of RSW can effectively respond to the mechanical behavior in the three stages (the elastic stage R_1_, the plastic stage R_2_, and the failure stage R_3_). Therefore, it provides strong evidence for adopting the FEM of RSW with IGs to investigate the influence of the IG on the mechanical properties and failure behaviors of RSW with similar thicknesses. The peak load of different group tensile shear specimens is shown in Table 3. The effect of the IG on the peak loads of tensile shear specimens is obvious. For the specimens of two types of plates with similar thicknesses, the peak load values decrease with the increment in the IG. The slope of the elastic stage R_1_ of the tensile shear RSW’s specimens decreases with the increase in the IG. This indicates that the warped deformation of RSW weakens the tensile strength with the increase in the IG. In the plastic stage R_2_, the bearing capacity of RSW also decreases with the increase in IG.

### 3.3. Peak Loads of FEM of RSW with IGs

**Figure 9:**
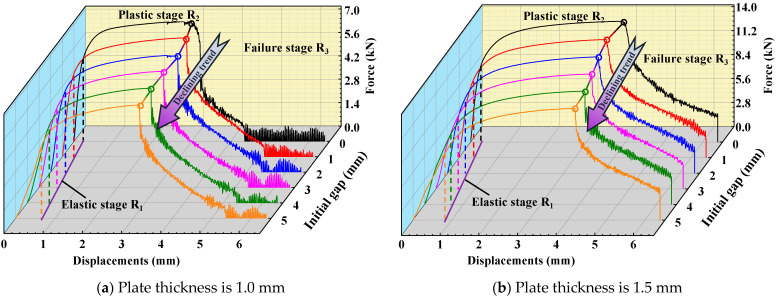
The force and displacement curves obtained from the simulation of RSW with different IGs.

In this paper, the refined FEMs of RSW with six different IGs are established to investigate the mechanical behaviors. The force and displacement curves are acquired from the simulation of RSW with six different IGs, as described in Figure 9. It is obvious that the peak load is gradually reduced with the increment in the IGs. With the increase in the IG, the characteristics of the elastic stage are gradually weakened. And in the plastic stage, the bearing capacity of RSW also decreases almost linearly with the increase in IG. This indicates that the IG causes a certain plastic deformation around the RSW, which reduces the strength of the RSW. However, the IG does not cause obvious crack propagation around the RSW. In the failure stage, the force and displacement curves exhibit a sudden decrease, indicating that the crack has penetrated through the thickness of the sheet. It is more consistent that the cracks penetrate the plate thickness and cracks propagate around the RSW. The peak load reduction rates based on the ideal RSW (that is, the IG is 0 mm) increase with the increase in IG, as shown in Table 4. The peak load reduction rates based on the ideal RSW for a 1.5 mm thickness are both higher than that for a 1.0 mm thickness under the same IG. This suggests that the IG has a greater impact on the deformation of the RSW as the thickness increases.

## 4. Discussion

### 4.1. The Failure Behaviors of RSW with IGs

**Figure 10:**
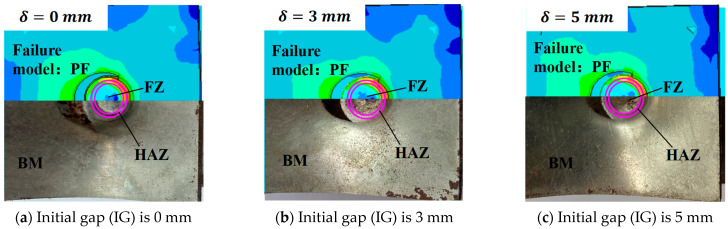
Pull-out fracture (PF) mode of RSW’s tensile shear specimens with three different IGs.

By analyzing the force and displacement curves of tensile tests, the deformation mechanism and failure behaviors of RSW can be further discussed. The failure behaviors of the tensile shear specimens are both PF mode, as shown in Figure 10. The stress concentration near the HAZ within the BM under the tensile shear load results in crack initiation and propagation since the hardness of BM is minimal in RSW zones. The microstructures of the RSW are changed during the welding process. Therefore, the tensile strength is changed with the microstructural change, as well as microhardness, as shown in Figure 5 and Figure 6. For the low-strength metal plate, the crack generally initiates and propagates near the HAZs with PF fracture [34]. The deformation around the RSW increases with the increase in the IG, leading to surface microcracks of the surroundings in the RSW. Therefore, the crack typically initiates from a minor surface defect and subsequently propagates along the boundary of the columnar grains in the thickness direction of the plate. As can be observed from the above, with the increase in IG, cracks are prone to occur in the BM near the HAZ, and the failure mode of the RSW is more inclined to PF. As plastic deformation accumulates and cracks develop, the elastic modulus of the material progressively diminishes [35], as shown in Figure 9. The peak loads are always identified in the plastic stage. Finally, the loads borne by specimens rapidly decreased in the failure stage with the increasing deformation of the BM near HAZ, as shown in Figure 8. Moreover, the mechanical performance of the low-carbon steel is improved with the increment in thickness.

### 4.2. The Prediction Model of Peak Load for RSW with IGs

**Figure 11:**
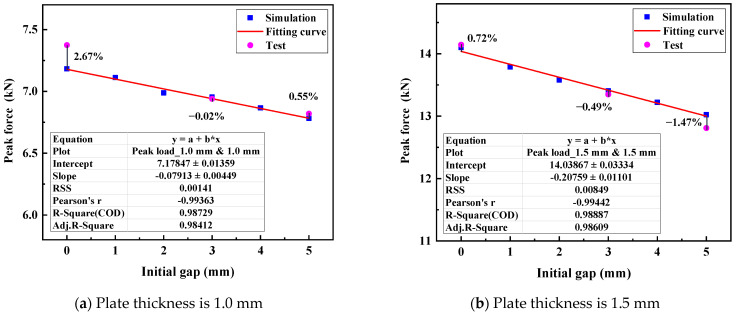
The peak loads of RSW with different IGs and fitting curves.

The effect of the IG on the peak load values of the tensile shear specimens is identified, as shown in Figure 10. The peak loads of the FEM of the RSW with the IGs are linearly fitted in this paper. The R-square for the plate thickness of 1.0 mm is almost 0.987, while the R-square for the plate thickness of 1.5 mm is almost 0.989. This indicates that the peak load values of the RSW decrease almost linearly with the increase in the IG. This also explains the principle of cumulative superposition in fracture mechanics. Therefore, the fitting formulas can be obtained in this paper, as shown in Equations (1) and (2). The fitting curves can effectively reveal the relationship between the IG and the peak load obtained from the FEM of RSW. The peak load of the RSW’s tensile shear specimens with the IGs (0 mm, 3 mm, and 5 mm) is shown in Figure 11. The errors between the fitting values and the test are relatively minor, where the maximum absolute error of RSW for a plate thickness of 1.0 mm is 2.67%, and the maximum absolute error of RSW for a plate thickness of 1.5 mm is 1.47%. It effectively verifies the relationship between the IG and the peak load. In addition, the accuracy of the FEM of RSW in responding to the mechanical behavior is discussed in Section 3.2.

(1) $$F_{peak}^{T_{1}}=7.17847-0.07913\delta$$

(2) $$F_{peak}^{T_{2}}=14.03867-0.20759\delta$$

where $F_{peak}^{T_{1}}$ is the peak load of RSW for the plate thickness of 1.0 mm, $F_{peak}^{T_{2}}$ is the peak load of RSW for the plate thickness of 1.5 mm, and 𝛿 is the IG.

Moreover, the comparison of the peak loads of the RSW with the IG among fitting values, simulation values, and test values are shown in Table 5. The absolute value of errors between the simulation values and fitting values (Sim. to Fit. Errors) are both within 0.5% for the plate thicknesses of 1.0 mm and 1.5 mm. The absolute values of errors between the test values and fitting values (Test. to Fit. Errors) are both within 3% for the plate thicknesses of 1.0 mm and 1.5 mm. This shows that the fitting curves can accurately predict the peak load of RSW with different IGs within 5 mm.

However, the peak load of the ideal RSW can be easily obtained in practical engineering applications. Furthermore, the peak load of the ideal RSW is typically utilized as the criterion for determining whether the RSW is qualified. The prediction model in Equations (1) and (2) is not of high applicability. Therefore, the fitting formulas need to be further adjusted to improve its applicability. The first terms in Equations (1) and (2) are approximate to the peak load of the IG of 0 mm for the plate thicknesses of 1.0 mm and 1.5 mm. Therefore, to enhance the applicability of the prediction model and more accurately describe the relationship between the peak load and IG, an influence factor f(T) is introduced, as defined in Equation (3). In addition, the prediction formulas in Equations (1) and (2), respectively, for the plate thicknesses of 1.0 mm and 1.5 mm are normalized as shown in Equation (4).

(3) $$f(T)=kT^{n}$$

(4) $$F_{peak}=F_{\delta =0}-f(T)\delta ,\left\{\begin{array}{c} f(T=1)=0.07913\\ f(T=1.5)=0.20759 \end{array}\right.$$

where $F_{peak}$ is the peak load of RSW, $F_{\delta =0}$ is the peak load of RSW with the IG of 0 mm, that is, the peak load of the ideal RSW, f(T) is the influence factor, T is the thickness of the plate, k is a constant coefficient, and n is a power exponent.

Through simultaneous equations, k and n are calculated as follows:

(5) $$\begin{array}{l} k=0.07913\\ n=2 \end{array}$$

Therefore, Equation (3) can be written as

(6) $$F_{peak}=F_{\delta =0}-0.07913T^{2}\delta$$

**Figure 12:**
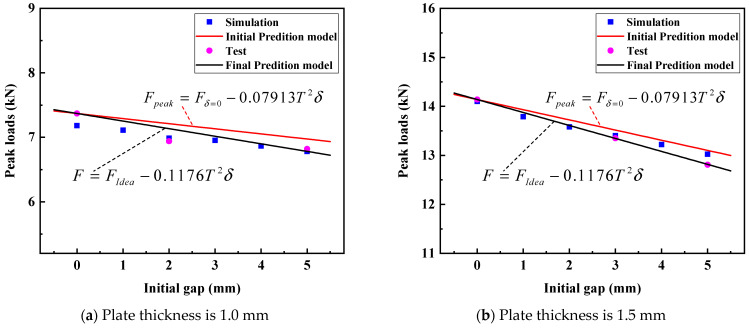
The prediction model of RSW with different IGs.

Due to the peak load of the ideal RSW being easily obtained by the specimen of the ideal RSW, the peak load of the test is selected as the value of $F_{\delta =0}$. In this paper, the $F_{\delta =0}$ values are 7.37 kN and 14.14 kN, respectively for the plate thicknesses of 1.0 mm and 1.5 mm. The comparison of the peak loads of RSW among the initial predicted values, simulation values, and test values is shown in Table 6. The maximum absolute errors between the simulation value and the predicted value (Sim. to Initial Pre. Errors) are 3.10% and 1.70%, respectively, for the plate thicknesses of 1.0 mm and 1.5 mm. The maximum absolute errors between the test values and the initial predicted values are 2.70% and 3.32%, respectively, for the plate thicknesses of 1.0 mm and 1.5 mm. However, the simulation values and test values for RSW with IGs are both relatively lower than the initial predicted curve, as shown in Figure 12 This phenomenon reveals that the initial prediction model presents an overall high trend, and its rationality is relatively insufficient. Therefore, it is necessary to further adjust and optimize the prediction model to improve the accuracy and reliability of the prediction results.

To improve the accuracy of predictions, the influence factor f(T) needs to be further adjusted. By optimizing the influential factor f(T), the error between the simulation and prediction results of the peak load in RSW with different IGs for two different sheet thicknesses is minimized. First, two prediction model functions for the plate thicknesses of 1.0 mm and 1.5 mm, respectively, are defined, as shown in Equation (7). The influence factor f(T) is *h* in Equation (7). Then, *Errors*1 and *Errors*2 between the simulated and predicted values for the plate thicknesses of 1.0 mm and 1.5 mm, respectively, are defined, as shown in Equations (8) and (9). In the error functions, the reference values $F_{peak1\delta }^{T_{1}}$ and $F_{peak2\delta }^{T_{2}}$ are shown in Equation (10). In this paper, based on the numerical optimization of the L-BFGS-B algorithm, the influence factor f(T) value that minimizes the sum of *Errors*1 and *Errors*2 is found. The optimization objectives and boundary conditions of the optimization algorithm are shown in Equation (11).

(7) $$\left.\begin{array}{l} F_{peak\delta }^{T_{1}}(h)=7.37-hT_{1}^{2}\delta \\ F_{peak\delta }^{T_{2}}(h)=7.37-hT_{2}^{2}\delta \end{array}\right\},\delta =\left[0,1,2,3,4,5\right]$$

(8) $$Errors1(h)=\sum_{\delta =1}^{5}\left|\frac{F_{peak\delta }^{T_{1}}(h)-F_{peak1\delta }^{T_{1}}}{F_{peak1\delta }^{T_{1}}}\right|\times 100$$

(9) $$Errors2(h)=\sum_{\delta =1}^{5}\left|\frac{F_{peak\delta }^{T_{2}}(h)-F_{peak2\delta }^{T_{2}}}{F_{peak2\delta }^{T_{2}}}\right|\times 100$$

(10) $$\left\{\begin{array}{l} F_{peak1\delta }^{T_{1}}=\left[7.182,7.112,6.988,6.954,6.866,6.782\right]\\ F_{peak2\delta }^{T_{2}}=\left[14.101,13.788,13.581,13.403,13.221,13.024\right] \end{array}\right.$$

(11) $$\begin{array}{l} \underset{h}{\min}J(h)=Errors1(h)+Errors2(h)\\ st:0\leq h\leq 2 \end{array}$$

**Figure 13:**
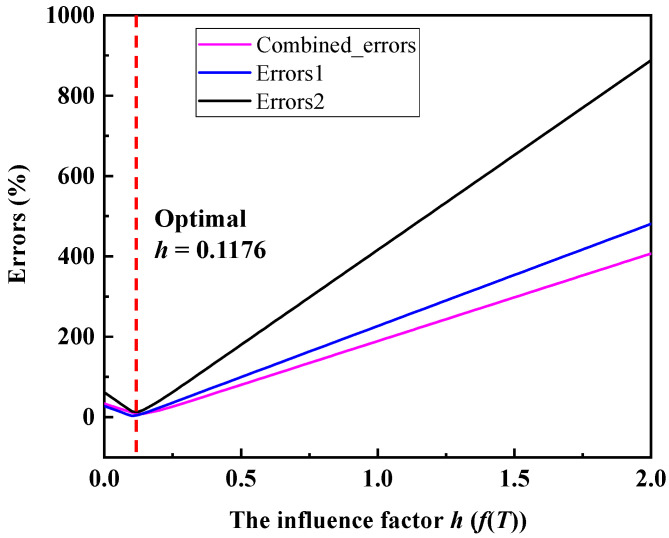
The errors minimization process for *h*.

Through the optimization algorithm, the *h* value for the combination error minimization is obtained as 0.1176, as shown in Figure 13. Therefore, the prediction model can be written as Equation (12). The variation in *Errors*1 and *Errors*2 in the optimization process can be seen in Figure 12.

(12) $$F_{peak}=F_{Idea}-0.1176T^{2}\delta$$

where $F_{Idea}$ is the peak load of the ideal RSW, that is, the peak load of RSW with the IG of 0 mm $F_{\delta =0}$.

**Figure 14:**
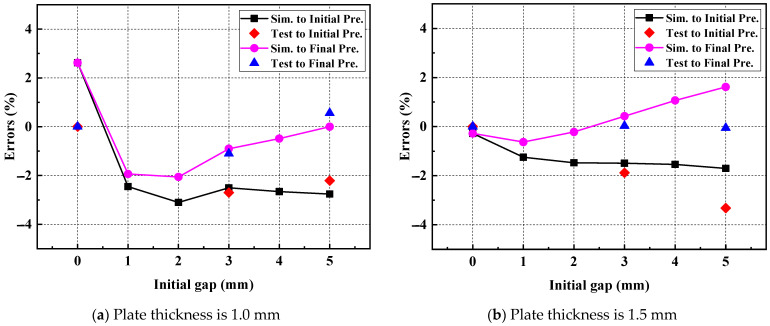
The errors of the simulation and test to the adjusted prediction model of RSW with different IGs.

As shown in Figure 12, the final prediction model can be better applied to simulation and test values than the initial prediction model. This shows that the final prediction model is suitable for predicting peak loads of RSW with different IGs. The errors of the simulation to the final predicted values (Sim. to Final Pre. Errors) and the test to the final predicted values (Test to Final Pre. Errors) are displayed in Table 7. The maximum absolute errors between the simulation and the predicted value are down to 2.62% and 1.62%, respectively, for the plate thicknesses of 1.0 mm and 1.5 mm. The maximum absolute errors between the test and the predicted values are down to 1.10% and 0.06%, respectively, for the plate thicknesses of 1.0 mm and 1.5 mm. The final prediction model reduces the errors of the simulation and the test to the final predicted values and improves the prediction accuracy, as shown in Figure 14. This indicates that the final prediction model can sufficiently predict the peak loads of RSW with different IGs.

## 5. Conclusions

The influence of the IG on the mechanical properties and failure behaviors of RSW with similar thicknesses is investigated in this paper. The peak loads of the RSW under the tensile shear loads with similar thicknesses are linearly decreased with the increase in the IG. The modulus of the elastic stage R1 decreases as well, and the plastic stage becomes smaller and smaller. The failure mode of RSW with the IG is both PF, and the failure location is in the BM near HAZ. To quantify the effect of the IG on the mechanical behavior of the RSW, a prediction model is introduced to predict the peak load of RSW with different IGs. Then, the prediction model is locally optimized through the L-BFGS-B optimization algorithm. It accurately predicts the peak load of the RSW with various IGs, with errors of no more than 3%. Based on the prediction model, the relationship among the peak load values of the tensile shear specimens, the IG, and the failure behavior of the RSW is revealed. This offers theoretical guidance for the prediction of the mechanical properties of RSW with such an IG.

## Figures and Tables

**Table 1:** Plate thickness and IG of the RSW’s tensile shear specimens.

Upper Plate Thickness T_1_	Lower Plate Thickness T_2_	IG 𝛿
1.0 mm	1.0 mm	0 mm
		3 mm
		5 mm
1.5 mm	1.5 mm	0 mm
		3 mm
		5 mm

**Table 2:** The average hardness values of three zones in RSW with three different IGs.

Categories	IG 𝛿 = 0 mm	IG 𝛿 = 3 mm	IG 𝛿 = 5 mm	Average
BM	120.01 HV	115.70 HV	125.43 HV	120.38 HV
HAZ	176.45 HV	175.90 HV	174.52 HV	175.62 HV
FZ	218.67 HV	209.94 HV	223.72 HV	217.44 HV

**Table 3:** The peak loads of tensile shear RSW’s specimens with three different IGs.

Types	IG 𝛿 = 0 mm	IG 𝛿 = 3 mm	IG 𝛿 = 5 mm
T_1__1.0 mm and T_2__1.0 mm	7.37 kN	6.94 kN	6.82 kN
T_1__1.5 mm and T_2__1.5 mm	14.14 kN	13.35 kN	12.81 kN

**Table 4:** The peak loads of tensile shear RSW’s simulation with different IGs.

Types	T_1__1.0 mm and T_2__1.0 mm		T_1__1.5 mm and T_2__1.5 mm	
	Peak Load (kN)	Reduction Rate (%)	Peak Load (kN)	Reduction Rate (%)
IG 𝛿 = 0 mm	7.182	0.00	14.101	0.00
IG 𝛿 = 1 mm	7.112	0.97	13.788	2.22
IG 𝛿 = 2 mm	6.988	2.71	13.581	3.69
IG 𝛿 = 3 mm	6.954	3.18	13.403	4.95
IG 𝛿 = 4 mm	6.866	4.39	13.221	6.24
IG 𝛿 = 5 mm	6.782	5.57	13.024	7.64

**Table 5:** Comparison of the peak loads among fitting values, simulation values, and test values.

Types		𝛿 = 0 mm	𝛿 = 1 mm	𝛿 = 2 mm	𝛿 = 3 mm	𝛿 = 4 mm	𝛿 = 5 mm
T_1__1.0 mm and T_2__1.0 mm	Fitting values (kN)	7.178	7.099	7.020	6.941	6.862	6.783
	Simulation values (kN)	7.182	7.112	6.988	6.954	6.866	6.782
	Sim. to Fit. Errors	0.05%	0.18%	0.46%	0.19%	0.06%	0.01%
	Test values (kN)	7.37	--	--	6.94	--	6.82
	Test to Fit. Errors	2.67%	--	--	0.02%	--	0.55%
T_1__1.5 mm and T_2__1.5 mm	Fitting values (kN)	14.039	13.831	13.623	13.416	13.208	13.001
	Simulation values (kN)	14.101	13.788	13.581	13.403	13.221	13.024
	Sim to Fit. Errors	0.44%	0.31%	0.31%	0.10%	0.10%	0.18%
	Test values (kN)	14.14	--	--	13.35	--	12.81
	Test to Fit. Errors	0.72%	--	--	0.49%	--	1.47%

**Table 6:** Comparison of the peak loads among the initial predicted values, simulation values, and test values.

Types		𝛿 = 0 mm	𝛿 = 1 mm	𝛿 = 2 mm	𝛿 = 3 mm	𝛿 = 4 mm	𝛿 = 5 mm
T_1__1.0 mm and T_2__1.0 mm	Initial Pre. values (kN)	7.37	7.291	7.212	7.132	7.053	6.974
	Simulation values (kN)	7.182	7.112	6.988	6.954	6.866	6.782
	Sim. to Initial Pre. Errors	2.62%	2.45%	3.10%	2.50%	2.66%	2.76%
	Test values (kN)	7.37	--	--	6.94	--	6.82
	Test to Initial Pre. Errors	0	--	--	2.70%	--	2.21%
T_1__1.5 mm and T_2__1.5 mm	Initial Pre. values (kN)	14.14	13.962	13.784	13.606	13.428	13.250
	Simulation values (kN)	14.101	13.788	13.581	13.403	13.221	13.024
	Sim. to Initial Pre. Errors	0.28%	1.25%	1.47%	1.49%	1.54%	1.70%
	Test values (kN)	14.14	--	--	13.35	--	12.81
	Test to Initial Pre. Errors	0	--	--	1.88%	--	3.32%

**Table 7:** Comparison of the peak loads among final predicted values, simulation values, and test values.

Types		𝛿 = 0 mm	𝛿 = 1 mm	𝛿 = 2 mm	𝛿 = 3 mm	𝛿 = 4 mm	𝛿 = 5 mm
T_1__1.0 mm and T_2__1.0 mm	Final Pre. values (kN)	7.37	7.252	7.135	7.017	6.899	6.782
	Simulation values (kN)	7.182	7.112	6.988	6.954	6.866	6.782
	Sim. to Final Pre. Errors	2.62%	1.94%	2.06%	0.90%	0.49%	0%
	Test values (kN)	7.37	--	--	6.94	--	6.82
	Test to Final Pre. Errors	0	--	--	1.10%	--	0.56%
T_1__1.5 mm and T_2__1.5 mm	Adjusted values (kN)	14.14	13.875	13.611	13.346	13.082	12.817
	Simulation values (kN)	14.101	13.788	13.581	13.403	13.221	13.024
	Sim. to Final Pre. Errors	0.28%	0.63%	0.22%	0.43%	1.07%	1.62%
	Test values (kN)	14.14	--	--	13.35	--	12.81
	Test to Final Pre. Errors	0	--	--	0.03%	--	0.06%

## Data Availability

Data will be made available on request.
